# Genetic diversity, reproductive performance, and genetic enhancement strategies in Huang-Huai goats

**DOI:** 10.3389/fgene.2025.1549051

**Published:** 2025-03-24

**Authors:** Kai Quan, Huibin Shi, Caihong Wei, Jun Li, Kun Liu, Huihua Wang, Wei Sun, Haoyuan Han

**Affiliations:** ^1^ College of Animal Science and Technology, Henan University of Animal Husbandry and Economy, Zhengzhou, Henan, China; ^2^ Institute of Animal Science, Chinese Academy of Agricultural Sciences, Beijing, China; ^3^ College of Animal Science and Technology, Yangzhou University, Yangzhou, China

**Keywords:** Huang-huai goat, hereditary, reproduction, intersexuality, PIS

## Abstract

The Huang-huai goat, indigenous to China’s Huang-Huai Plain, is celebrated for its exceptional reproductive capacity, succulent meat, and superior leather qualities. The Huang-huai goat’s reproductive characteristics, genetic diversity, and the genetic underpinnings of intersexuality, aiming to inform conservation efforts and genetic resource management. Our study at *the Huang-huai Goat Science and Technology R&D Center* monitored 600–800 female goats and 16–24 male goats from June 2020 to May 2022, adhering to NIH guidelines and with ethical approval from *Henan University of Animal Husbandry and Economics*. Our findings indicate that these goats exhibit a year-round estrus cycle averaging 19–23 days, a gestation period of 146–150 days, and an average litter size of 2.74, with an annual reproduction rate of 418.96% and a weaning survival rate of 94.75%. Transcriptome sequencing identified eleven candidate genes associated with multiple offspring, including *PTX3*, *MMP13*, and *NR4A1*, which play roles in organ development and hormonal regulation. SNP analysis revealed specific genotypes in *GJB6* and *PRKAA1* linked to higher lambing numbers, offering molecular markers for selective breeding. The study also highlighted the role of the Polled Intersex Syndrome (PIS) locus in causing both hornless and intersexual traits, emphasizing the importance of genetic screening for maintaining breed health and productivity. The genetic resources of the Huang-huai goat, recognized as a national geographical indication product, are vital for the livestock industry and require strategic conservation for sustainable development. This review highlights the importance of preserving and utilizing the genetic resources of the Huang-huai goat to enhance its contribution to agriculture.

## 1 Introduction

Nestled between 33° and 35.5°N latitude and 113° and 119°E longitude, the Huang-Huai Plain boasts a climate of humidity and ample sunlight, alongside an abundance of natural resources that have historically established it as the cradle of ancient Chinese civilization (Zhou et al., 2014; Hu et al., 2021). The region’s advantageous irrigation systems and substantial agricultural byproducts, particularly crop straw, make it an ideal hub for industrial-scale agriculture and animal husbandry, and it has been duly recognized as a pivotal grain-producing area in China (Xiao et al., 2022). This fertile landscape is especially hospitable to goat farming.

The Huang-Huai goat, also known as the Huai goat, derives its name from the Huang-Huai Plain where it has its origin (Zhou et al., 2020). There are over 10 million of these goats dispersed across the eastern part of Henan, the northern regions of Anhui, and the northern areas of Jiangsu (Li et al., 2022). These medium-sized goats possess short white hair and pink skin. They can be classified into horned (accounting for 43.03%) and hornless (accounting for 56.97%) varieties (Hua et al., 2008). On average, adult male goats weigh 49.08 kg, while adult female goats weigh 37.75 kg (Pan et al., 2011). Since 2000, excessive crossbreeding of Huang-huai goats with Boer goats has led to the degradation of their germplasm advantages–including high fertility, superior meat quality, and premium leather traits–resulting in diminished reproductive efficiency and compromised market reputation (Han et al., 2022). Unrestricted hybridization has resulted in a deterioration in reproductive performance, as well as in the quality of meat and hides (Yang et al., 2021). In particular, the high occurrence rate of intersexuality has hindered the selection and propagation of the hornless trait, posing a threat to the future of this breed (Han et al., 2022; Yang et al., 2021).

To enhance the conservation and efficient use of Huang-Huai goat germplasm resources, the expert team of the *Henan agricultural research system of sheep and Goat Industry Technology (HARS-22–15)*, relying on *the Shenqiu Huai Goat Technology R*&*D Center*, has conducted a comprehensive and systematic assessment of its production performance over a period of 5 years. This review summarizes the reproductive characteristics and progress in genetic improvement of the Huang-Huai goat.

## 2 Genetic diversity and maternal origin of Huang-huai goats

### 2.1 Genetic diversity and its significance

Genetic diversity is a crucial indicator for the conservation of germplasm resources. Studies have revealed that the Huang-Huai goat, along with several other local breeds such as the Funiu white goat and Yaoshan white goat, exhibit relatively high genetic polymorphism. This indicates that these breeds possess abundant genetic resources and strong adaptability to the environment.

### 2.2 Maternal origin analysis

Maternal origin analysis has demonstrated that these breeds mainly originated from the Markhor. There are two maternal lineages, namely lineage A and lineage B, which reveals the complex evolutionary history and genetic background of the Huang-Huai goat.

### 2.3 Mitochondrial DNA research

Research on mitochondrial DNA (mtDNA) has provided direct evidence for the maternal origin of the Huang-Huai goat. Studies on the mitochondrial D-loop region of domestic goats in the central region have shown that the maternal origin of these breeds is the Markhor. Moreover, the majority of individuals are from lineage A, while a small number are from lineage B, further confirming the hypothesis of multiple maternal origins of the Huang-Huai goat.

## 3 Reproductive performance of Huang-huai goats

The research on the reproductive performance of Huang-Huai goats was carried out at *the Huang-Huai Goat Science and Technology R*&*D Center*, which is dedicated to the study, demonstration, and promotion of this particular breed. From June 2020 to May 2022, the center accommodated 600–800 female goats and 16–24 male goats of the Huang-Huai breed. All the animals were taken care of in strict accordance with the guidelines stipulated by the National Institutes of Health regarding the care and use of experimental animals. Moreover, all the procedures and experiments were duly approved by *the Animal Care and Use Committee of Henan University of Animal Husbandry and Economics.*

### 3.1 Study design and data collection

A reproduction test was carried out on Huang-Huai goats. Daily observations and data collection were made concerning estrus performance, breeding status, heat duration, and male goat semen quality. Data filtering was implemented to exclude any values that deviated beyond the mean ± standard deviation range for each trait. Statistical analysis was then performed on various parameters, including the first estrus period, estrus duration, breeding cycle, average litter size, lamb weaning survival rate, average number of weaned lambs per female per year, and male goat semen quality.

### 3.2 Puberty and reproductive characteristics

Male lambs display signs of puberty through behaviors such as sniffing the vulva of female goats, chasing them upon detecting estrus (sexual olfactory reflex), barking after olfactory perception, penile erection, mounting, and ejaculation (Quan et al., 2020). The average age of puberty for male lambs ranges from 75 to 83 days, with an average weight of 15–23 kg. Female lambs exhibit symptoms of puberty like mental excitement, loss of appetite, tail erection, and vulvar swelling accompanied by secretions, although they do not permit male goats to mount them. The average age and weight at puberty for female goats are 63–84 days and 14–22 kg respectively. They reach sexual maturity at 4–5 months of age, with a weight of 18–25 kg. Huang-Huai goats show a relatively higher proportion of estrus in autumn and winter, yet they can experience estrus throughout the year, with an estrus cycle spanning from 18.3 to 22.8 days. The gestation period is 146–150 days, and the average lambing interval is 238.34 days. The average litter size is 2.74, and the annual reproduction rate amounts to 418.96% (Table 1). The weaning survival rate is 94.75%, with an average of 3.97 weaned lambs per female per year (Table 2).

**TABLE 1:** The estrus and lambing of female goats in the month from June 2020 to May 2022.

Months	Female goats (n)	Number of estrus goats (n)	Number of delivery female goats (n)	Number of lambs born (n)	Number of weaned lambs (n)
January	1,242	165	152	433	421
February	1,233	144	187	537	442
March	1,238	130	205	565	426
April	1,267	143	196	462	468
May	1,241	167	174	488	457
June	1,302	168	151	435	454
July	1,271	191	133	361	421
August	1,236	166	121	302	386
September	1,265	205	136	385	320
October	1,283	224	154	443	371
November	1,303	219	149	402	413
December	1,242	191	172	467	424
Total	-	2,113	1930	5,280	5,003

The data are the sum of 2 years, the number of female goats was the statistics at the end of last month, and the number of total female goats was average of 2 years.

**TABLE 2:** Statistics of different litter size.

Item	Number of lambing female goats (n)	Number of lambs (n)	Weaning lamb number (n)	Lamb weaning survival rate (%)
Single	43	43	43	100.00
Double	476	952	950	99.79
Triple	1,364	4,092	3,871	94.60
Quadruple	42	168	124	73.81
Quintuple	5	25	15	60.00
Total	1930	5,280	5,003	94.75

### 3.3 Semen quality and reproduction seasonality

Huang-Huai male goats are capable of reproducing throughout the year. Their semen quality reaches its peak in autumn and winter and deteriorates in summer (Table 3). The average ejaculation volume is 1.01 ± 0.11 mL, with an average sperm motility of 84.49% ± 2.26% and a sperm concentration of (21.52 ± 6.86) × 10^8^/mL. The defective sperm rate is 12.16% ± 2.31%, and the semen is usually milky white.

**TABLE 3:** Semen quality of male goats in different seasons.

Season	Number of male goats(n)	Ejaculate volumes (mL)	Sperm motility rate (%)	Sperm concentration (10^8^/mL)	Defective sperm rate (%)
Spring	26	1.01 ± 0.11	81.12 ± 3.22	21.53 ± 7.29	13.42 ± 2.92
Summer	20	0.88 ± 0.17	80.73 ± 3.59	16.59 ± 8.18	14.34 ± 2.67
Autumn	36	1.23 ± 0.08	89.14 ± 1.21	25.14 ± 6.34	10.12 ± 1.31
Winter	31	1.19 ± 0.12	84.33 ± 1.83	22.83 ± 5.61	11.42 ± 2.71
Average	-	1.01 ± 0.11	84.49 ± 2.26	21.52 ± 6.86	12.16 ± 2.31

### 3.4 Reproductive performance and breeding efficiency

Reproductive performance is a crucial index for evaluating goat production levels, especially in large-scale farming systems. In comparison with other Chinese local breeds and introduced breeds like Boer goats (Wang et al., 2020), Huang-Huai goats reach puberty and sexual maturity at an earlier stage. They can reproduce throughout the year with minimal seasonal impact, attaining an average of 1.57 pregnancies per year, and each female can wean approximately 3.97 lambs annually.

The early sexual maturity and high reproductive performance of Huang-Huai goats confer significant benefits upon breeders. It enables early breeding and shorter generation intervals, which can boost productivity and potentially augment profits (Chu et al., 2011). Although the breed’s inherent characteristics contribute to its early reproductive development, optimal management and a balanced diet are indispensable for maintaining the peak reproductive performance in goat farming.

## 4 Candidate genes for multiple lambs in Huang-huai goats

To understand the genetic basis of multiple lambs in Huang-Huai goats, 3-year-old healthy female goats with similar body conditions were selected and grouped based on their reproductive records: a single-lamb group (24 females, averaging one lamb per birth) and a multi-lamb group (24 females, averaging over two lambs per birth). Transcriptome sequencing was used to build mRNA libraries for both groups to identify differentially expressed genes (DEGs), followed by functional enrichment analysis and verification with real-time fluorescence quantitative PCR. Blood genomic DNA from goats with different reproductive performances was extracted. Based on preliminary transcriptome analysis and literature review, *GJB6* (gap junction protein beta 6) and *PRKAA1* (AMP-activated protein kinase catalytic subunit alpha-1) were prioritized for SNP analysis due to their established roles in follicular development and metabolic regulation of reproduction in other ruminants (Domínguez-Ruiz et al., 2024; Wang et al., 2020; Xiao et al., 2020). Candidate genes related to multiple lambs were SNP scanned and genotyped, and the correlation with high lambing frequencies was analyzed. Primers for *GJB6* (forward: 5′-CAG​GTG​CTG​GAC​TTC​ATC​CT-3′; reverse: 5′-TGG​CAA​TGT​CAC​AGA​GGA​CA-3′) and *PRKAA1* (forward: 5′-GCT​GGA​CCT​CAA​CCT​GAT​GA-3′; reverse: 5′-AGC​CAC​AGG​GTC​TTC​ATG​GT-3′) were designed using Primer-BLAST (NCBI).

**FIGURE 1:**
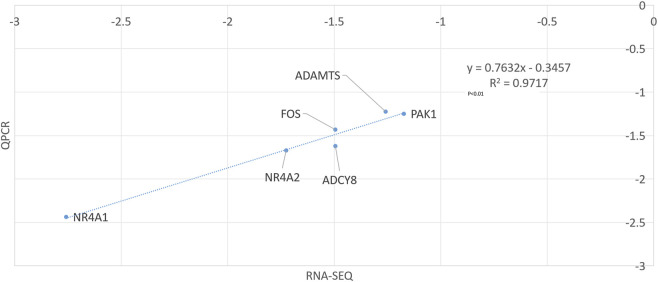
qPCR validation of gene expression levels in single- and multi-lamb groups. Using *GAPDH* as the reference gene, qRT-PCR analysis revealed significant downregulation of *ADCY8, FOS, PAK1, NR4A2, ADAMTS*, and *NR4A1* in the ovaries of multiparous Huang-Huai goats compared to uniparous goats, consistent with RNA-seq results. A strong Pearson correlation (R^2^ = 0.9717, *P* < 0.01) between RNA-seq and qRT-PCR fold-change values confirmed the high reliability of RNA-seq data (Han et al., 2023b).

Eleven candidate genes related to lambing number in Huang-Huai goats were found by comparing the transcriptomes of the two groups (Gawat et al., 2023): *PTX3*, *MMP13*, *PAK1*, *ADAMTS1*, *COL1A2*, *CCN1*, *SLC4A10*, *FOS*, *NR4A1*, *NR4A2*, and *ADCY8*. These genes are involved in animal organ development and hormone secretion in the endocrine system (Yao et al., 2023), and qPCR validation confirmed differential expression patterns (*P* < 0.05) between single- and multi-lamb groups (Figure 1).

Specific primers were designed for the *GJB6* and *PRKAA1* genes in Huang-Huai goats. After sequencing and genotyping, two synonymous mutations in *GJB6* and three SNP sites in the non-coding sequence of *PRKAA1* were detected. The TC genotype of g.33691489 T>C and the AT genotype of g.33693395 A>T in *PRKAA1* were associated with a higher number of lambs (Table 4). Haplotype analysis further revealed that the TCA haplotype (comprising SNPs g.33691489 T>C, g.33693100 T>C, and g.33693395 A>T) was significantly associated with increased prolificacy (χ^2^ = 8.24, *P* = 0.004), aligning with linkage disequilibrium patterns in the *PRKAA1* locus.

**TABLE 4:** The association analysis between SNPs of *GJB6*/*PRKAA1* gene and lambing numbers of Huai goats (average ±standard deviation).

Gene	SNP	Genotype	No.	Lambing number
*GJB6*	g.50694819 C>T	CC	103	1.670 ± 0.103
		CT	16	1.711 ± 0.169
		TT	3	1.654 ± 0.371
	g.50694816 T>C	CC	8	1.747 ± 0.223
		CT	42	1.710 ± 0.165
		TT	72	1.578 ± 0.168
*PRKAA1*	g.33691489 T>C	TT	25	1.600 ± 0.139^AB^
		TC	58	1.953 ± 0.125^A^
		CC	39	1.350 ± 0.169^B^
	g.33693100 T>C	TT	103	1.557 ± 0.134
		TC	16	1.738 ± 0.205
		CC	3	1.000 ± 0.400
	g.33693395 A>T	AA	34	1.492 ± 0.250^B^
		AT	63	1.780 ± 0.151^A^
		TT	25	1.321 ± 0.170^B^

Haplotype frequencies were analyzed using PHASE, v2.1, and association tests were performed with PLINK v1.9.

Different uppercase letters indicate extremely significant difference (P < 0.01), same letters or not marked indicate no significant difference (P > 0.05) (Han et al., 2023a).

Transcriptome sequencing helps find DEGs among different phenotypes. Some genes related to follicular development, ovulation, and steroid hormone synthesis are expressed in ovarian tissues of Huang-Huai goats (Ceccobelli et al., 2023). For example, *MMP13* is important for follicle formation and ovulation, and *ADAMTS1* promotes follicular development and maturation. *NR4A1* and *NR4A2* are involved in the ARH response related to reproductive function and may regulate lambing (McNulty et al., 2012).

SNPs are related to gene expression and individual trait differences. In Huang-Huai goats, the TC genotype at g.33691489 T>C and the AT genotype at g.33693359 A>T in *PRKAA1* are significantly associated with a higher lambing number (Wang et al., 2019). Haplotype analysis shows that the TCA haplotype is also related to increased lambing. These loci can be potential molecular markers for selecting multiple lambing traits.

## 5 Intersex syndrome in Huang-huai goats

Six intersex Huang-Huai goats were slaughtered to study their reproductive organ anatomy (Han et al., 2022; Yang et al., 2021). HE staining was performed on ovarian samples to histologically evaluate tissue structure, identify pathological changes (e.g., testicular-like lesions), and confirm the presence of intersex-specific anomalies (e.g., mixed ovarian/testicular tissues), which are critical for diagnosing PIS. Blood samples from 18 intersex and 45 normal goats (12 females and 23 males) were collected. PCR was used to amplify the SRY gene and detect the PIS deletion.

**FIGURE 2:**
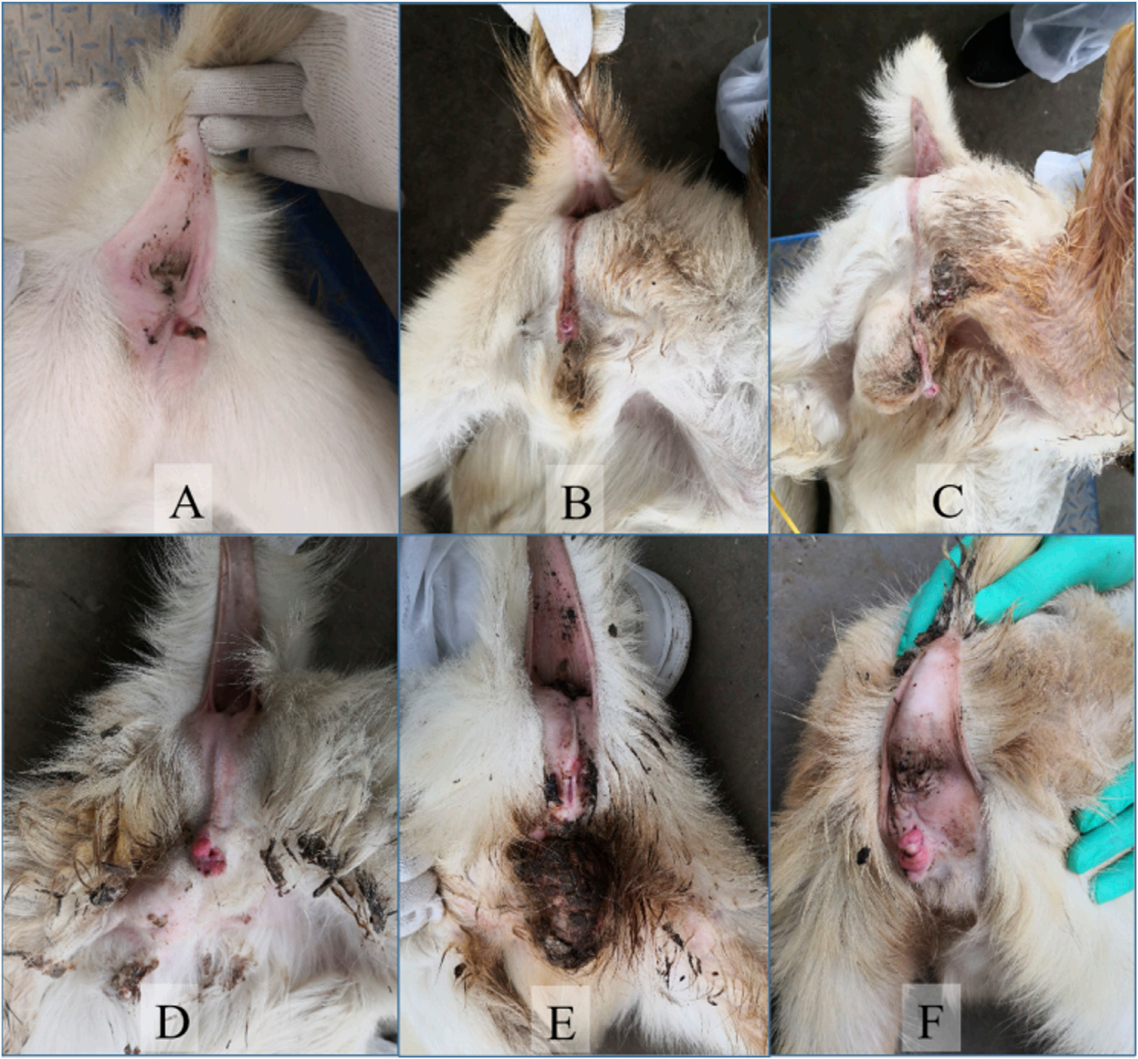
Observation of the external genitalia of intersexual Huang-huai goats. A-F represent external genitalia of intersexual Huang-huai goats. Intersexual Huang-huai goats have female reproductive organs, but with an enlarged, prominent clitoris. The vulva is smaller, and the clitoris resembles a penis. Some individuals may have seemingly male reproductive organs, but they are incompletely developed. Overall, their external genital morphology is intermediate between normal male and female goats (Yang et al., 2021).

**FIGURE 3:**
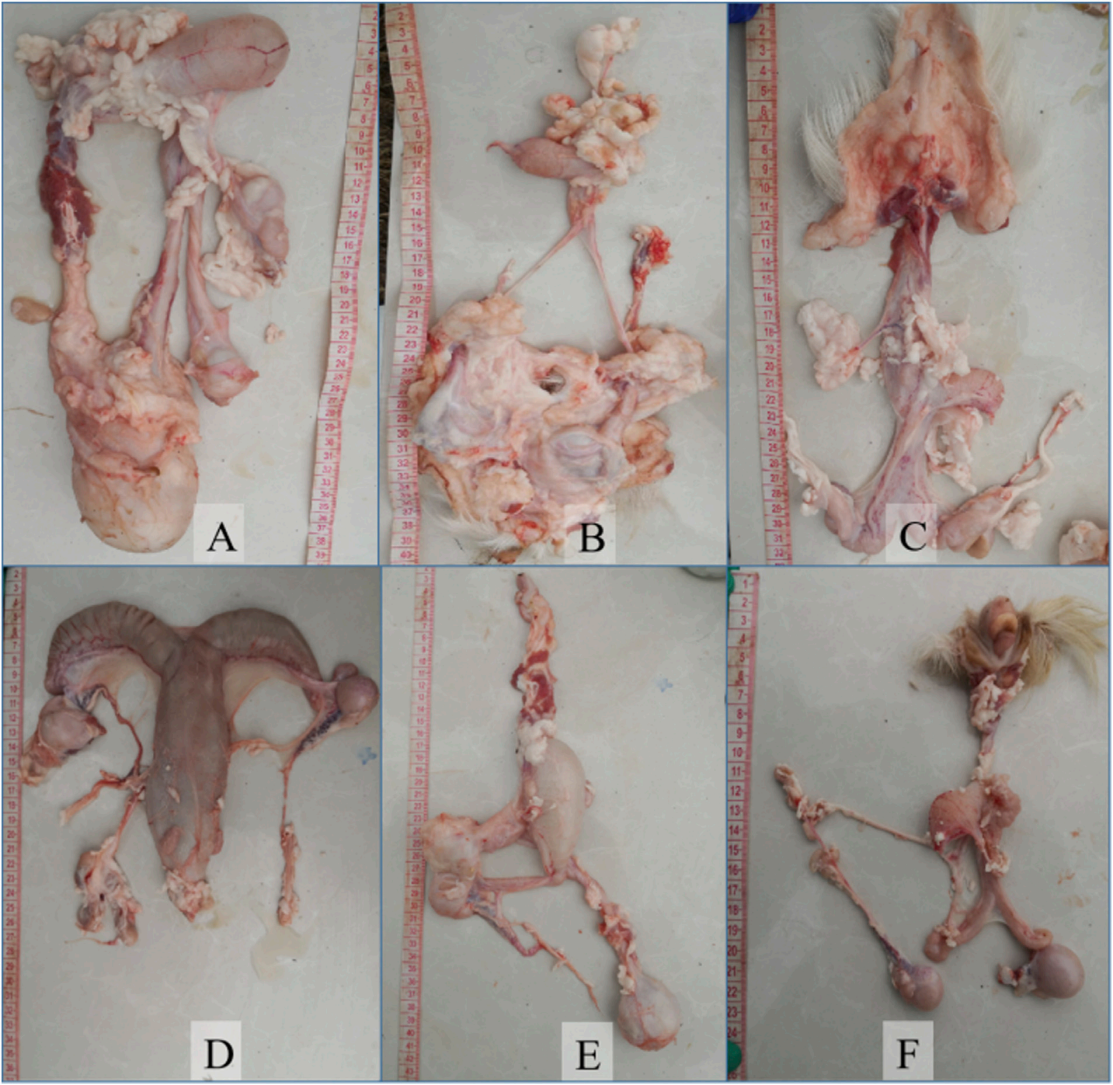
Observation of the internal genitalia of intersexual Huang-huai goats. A-F represent the gonads of intersexual Huang-huai goats. It showed significant morphological differences from typical female ovaries and are larger in size. Histologically, they show a mixed pattern, similar to ovarian tissue with testicular-like lesions or partially differentiated testicular structures. The reproductive system has well-defined female organs (uterus and vagina) but also underdeveloped male components (rudimentary ductal systems or incomplete spermatic cord formation), representing a transitional state between male and female reproductive organ development (Yang et al., 2021).

Externally, intersex goats had a smaller vulva. Some had testes but no vulva and had underdeveloped genitals causing dysuria (Figure 2). Internally, sample A had ovarian-like gonads larger than normal ovaries with follicular structures and swollen female reproductive ducts. Sample B had one large and one small gonad with different connections to genital tracts and a nearby penis-like structure. Samples C, D, E, and F had uneven-surfaced gonads, with the left gonad having a spermatic cord and a degenerated uterus (Figure 3).

SRY amplification showed 18 intersex goats had XX sex chromosomes. Using Monteagudo’s and Simon’s methods (Monteagudo et al., 2008; Simon et al., 2020), it was found that the 18 intersex goats had homozygous PIS deletions. Among 45 normal goats, 17 were PIS+/+ homozygous wild-type and 28 were PIS+/− heterozygous. Among 23 male goats, 8 were PIS+/+ and 15 (65.21%) were PIS+/− heterozygous. Among 22 female goats, 9 were PIS+/+ and 13 (59.09%) were PIS+/− heterozygous (Table 5).

**TABLE 5:** Distinguishing types of PIS between normal and intersex Huang-huai goats.

Genotype individual	Number	Gender	Individual number
PIS^+/+^	17	♀	9
		♂	8
PIS^+/−^	28	♀	13
		♂	15

In normal Huang-Huai goats, 62% carry PIS deletion, with 65.21% of male goats and 59.09% of female goats. Homozygous PIS deletion in offspring causes intersex traits. If carriers aren’t excluded, the intersex gene frequency will rise, reducing population reproduction and economic benefits. So, intersex detection in Huang-Huai goats is urgent. The study found that the PIS locus causes the hornless trait (autosomal dominant) and the intersexual trait (autosomal recessive) in Huang-Huai goats. The two traits are linked, with intersexuality usually in hornless goats and regulated by the PIS region (100 kb on chromosome 1q43, between *ATP1B* and *COP* genes, with a 11.7 kb fragment deletion causing the syndrome). The PIS region transcribes genes like *FOXL2*, *PISRT1*, and *PFOXic*.

## 6 Application of genetic improvement for Huang-huai goat

### 6.1 Enhancing reproductive performance through genetic selection

Our team has made significant strides in the genetic enhancement of the Huang-huai goat by optimizing the breeding core group for multiparous traits. Utilizing genomic tools, we have developed breeding chips that facilitate the identification of multiparous female goats. This technology has been applied across three conservation farms, leading to the establishment of a core breeding group comprising 1,200 goats. This targeted approach has accelerated the enrichment of multiparous genes, concentrating superior genetic traits within the core group and thereby enhancing the overall reproductive performance of the population.

### 6.2 Incorporating PIS testing to preserve genetic integrity

We have implemented PIS testing for male goats, a crucial step in maintaining the genetic health of the breed. This initiative involved screening 210 local breeding males, resulting in the exclusion of 134 individuals carrying the intersex gene. This proactive measure has substantially reduced the prevalence of intersex traits among Huang-huai goats, safeguarding the genetic integrity of the breeding population and preventing the spread of deleterious genes, which is vital for the sustainable development and reproductive vigor of the breed.

## 7 Discussion

This review comprehensively elucidates the genetic diversity, reproductive traits, and genetic enhancement strategies of Huang-Huai goats, providing critical insights for breed conservation and sustainable utilization. The high genetic polymorphism and dual maternal lineages (A and B) underscore the breed’s evolutionary resilience, aligning with findings in other indigenous Chinese breeds (Peng et al., 2024). Notably, Huang-Huai goats exhibit exceptional reproductive performance, including year-round estrus, a high annual reproduction rate (418.96%), and robust weaning survival (94.75%), surpassing many local and introduced breeds (Han et al., 2022). The identification of candidate genes (*PTX3, MMP13, PRKAA1*) and SNPs associated with prolificacy offers novel molecular markers for selective breeding, complementing prior studies on fecundity-related genes in goats (Han et al., 2022). However, functional validation of these genes and their regulatory mechanisms in ovulation and hormone synthesis warrants further investigation.

The linkage between the PIS locus and intersexuality highlights a critical challenge in breeding hornless variants. The high carrier rate (62%) of PIS deletions emphasizes the urgency of genetic screening to mitigate intersex traits, as proposed in Polled goat management (Yang et al., 2021). While this study establishes a framework for PIS detection, broader validation across diverse populations is essential to ensure applicability. Additionally, environmental and epigenetic factors influencing reproductive performance remain unexplored, representing a limitation of the current dataset (Guang-Xin et al., 2018).

Future efforts should focus on expanding genomic databases, integrating multi-omics approaches to unravel gene-environment interactions, and refining breeding chips for precision selection (Liu et al., 2024). Collaborative initiatives to disseminate genetic screening protocols and core breeding strategies will enhance breed purity and productivity. This work lays the foundation for leveraging Huang-Huai goats’ genetic potential, balancing conservation with agricultural innovation in China’s livestock sector.

## 8 Conclusion

The exploration, conservation, and strategic utilization of the Huang-Huai goat germplasm resources are essential for the protection of biodiversity and the advancement of new, distinctive breeds (Zhang et al., 2024; Jin et al., 2020). This study has conducted a thorough examination of the genetic diversity, maternal lineage, and reproductive performance of the Huang-Huai goats. By concentrating on candidate genes associated with multiple births, we have developed a specialized breeding chip for the Huang-Huai goats and have selected a core breeding population. Additionally, to address the intersex syndrome observed in the Huang-Huai goats, a targeted screening protocol has been implemented to identify and exclude male goats carrying intersex genetic markers. These efforts have substantially enhanced the technical infrastructure for the conservation and efficient utilization of the Huang-Huai goat germplasm resources.
